# Sex-Specific Associations of Brain-Derived Neurotrophic Factor and Cardiorespiratory Fitness in the General Population

**DOI:** 10.3390/biom9100630

**Published:** 2019-10-20

**Authors:** Marie-Lena Schmalhofer, Marcello R.P. Markus, Jan C. Gras, Juliane Kopp, Deborah Janowitz, Hans-Jörgen Grabe, Stefan Groß, Ralf Ewert, Sven Gläser, Diana Albrecht, Ina Eiffler, Henry Völzke, Nele Friedrich, Matthias Nauck, Antje Steveling, Stephanie Könemann, Kristin Wenzel, Stephan B. Felix, Marcus Dörr, Martin Bahls

**Affiliations:** 1 Department of Internal Medicine B, University Medicine Greifswald, 17475 Greifswald, Germanyjan.c.gras@googlemail.com (J.C.G.); nane.kopp@gmail.com (J.K.); stefan.gross1@uni-greifswald.de (S.G.); ewert@uni-greifswald.de (R.E.); stephanie.koenemann@uni-greifswald.de (S.K.); kristin.wenzel@uni-greifswald.de (K.W.); felix@uni-greifswald.de (S.B.F.); marcus.doerr@uni-greifswald.de (M.D.); 2 DZHK (German Centre for Cardiovascular Research), partner site Greifswald, 17475 Greifswald, Germany; voelzke@uni-greifswald.de (H.V.); nele.friedrich@med.uni-greifswald.de (N.F.); matthias.nauck@med.uni-greifswald.de (M.N.); 3 Department of Psychiatry and Psychotherapy, University Medicine Greifswald, 17475 Greifswald, Germany; deborah.janowitz@med.uni-greifswald.de (D.J.); hansj.grabe@med.uni-greifswald.de (H.-J.G.); 4 DZNE (German Centre for Neurodegenerative Diseases), partner site Greifswald, 17475 Greifswald, Germany; 5 Department of Internal Medicine, Vivantes Klinikum Spandau, 13407 Berlin, Germany; Sven.Glaeser@vivantes.de; 6 Institute for Community Medicine, University Medicine, 17475 Greifswald, Germany; diana.albrecht@inp-greifswald.de; 7 Leibniz Institute for Plasma Science and Technology, 17489 Greifswald, Germany; 8 Institute for Cell Biology and Anatomy, University Medicine Greifswald, 17487 Greifswald, Germany; ina.eiffler@uni-greifswald.de; 9 Institute of Clinical Chemistry and Laboratory Medicine, University Medicine Greifswald, 17475 Greifswald, Germany; 10 Department of Internal Medicine A, University Medicine Greifswald, 17475 Greifswald, Germany; antje.steveling@uni-greifswald.de

**Keywords:** BDNF, cardiorespiratory exercise capacity, cardiorespiratory fitness

## Abstract

The brain-derived neurotrophic factor (BDNF) was initially considered to be neuron-specific. Meanwhile, this neurotrophin is peripherally also secreted by skeletal muscle cells and increases due to exercise. Whether BDNF is related to cardiorespiratory fitness (CRF) is currently unclear. We analyzed the association of serum BDNF levels with CRF in the general population (Study of Health in Pomerania (SHIP-TREND) from Northeast Germany; *n* = 1607, 51% female; median age 48 years). Sex-stratified linear regression models adjusted for age, height, smoking, body fat, lean mass, physical activity, and depression analyzed the association between BDNF and maximal oxygen consumption (VO_2_peak), maximal oxygen consumption normalized for body weight (VO_2_peak/kg), and oxygen consumption at the anaerobic threshold (VO_2_@AT). In women, 1 mL/min higher VO_2_peak, VO_2_peak/kg, and VO_2_@AT were associated with a 2.43 pg/mL (95% confidence interval [CI]: 1.16 to 3.69 pg/mL; *p* = 0.0002), 150.66 pg/mL (95% CI: 63.42 to 237.90 pg/mL; *p* = 0.0007), and 2.68 pg/mL (95% CI: 0.5 to 4.8 pg/mL; *p* = 0.01) higher BDNF serum concentration, respectively. No significant associations were found in men. Further research is needed to understand the sex-specific association between CRF and BDNF.

## 1. Introduction

The brain-derived neurotrophic factor (BDNF) is a member of the neurotrophic family of growth factors. BDNF is essential for the differentiation, survival, and maintenance of neurons [1]. However, it is not neuron-specific, but is also found in and synthesized by endothelial [2] and skeletal muscle cells [3]. In the blood, BDNF is mostly bound and stored in platelets [4]. Furthermore, experimental studies showed the ability for BDNF to cross the blood–brain barrier in both directions [5].

Recent research demonstrated an association between low BDNF levels and an increased risk for cardiovascular diseases [6]. Moreover, low BDNF was associated with an increased incidence of coronary events and mortality in patients with angina pectoris [7], as well as in heart failure patients [8]. In contrast, higher levels of cardiorespiratory fitness (CRF) seem to be related to a lower risk of cardiovascular diseases [9]. Specifically, for every metabolic equivalent task increase in CRF, the hazards ratio for major adverse cardiovascular events decreased by 16% [10]. Since BDNF is secreted by contracting skeletal muscle cells, exercise and CRF may influence the synthesis of this neurotrophin [11]. This is supported by earlier studies, which demonstrated increased levels of BDNF in the peripheral blood after exercise training [12,13,14,15]. Thus, exercise training increases circulating BDNF. In contrast, previous studies reported an inverse correlation between CRF and BDNF [16,17,18,19]. A small trial with physically active males (*n* = 12) showed that step count, daily total energy expenditure, and movement-related energy expenditure were inversely correlated with BDNF levels [17]. This was supported by a small cross-sectional study of 44 subjects without overt cardiometabolic disease, which reported an inverse correlation between BDNF and estimated VO_2_peak (*r* = −0.352; *p* < 0.05) as well as physical activity (*r* = −0.428; *p* < 0.01) [16]. In untrained healthy Korean men, BDNF was also inversely correlated with VO_2_peak [18]. However, as far as we know, there is no consensus regarding the relationship between CRF and BDNF.

Given the paucity of literature discussing this relation, we aimed to analyze the associations of CRF with serum BDNF levels in a large sample from the population-based Study of Health in Pomerania (SHIP-Trend). Parameters for CRF were maximal oxygen uptake (VO_2_peak), VO_2_peak adjusted for body weight (VO_2_peak/kg), and oxygen uptake at the anaerobic threshold (VO_2_@AT). Age, smoking, body composition, physical activity, and depression [20,21] influence both BDNF [22,23,24] and CRF [25,26]. Hence, these parameters were included as confounders. Further, very recently, BDNF was identified as sexually dimorphic neurotrophin [27,28].

## 2. Materials and Methods

### 2.1. Study Population

SHIP-Trend is a cross-sectional population-based study in Northeast Germany. From 2008 to 2012, 8826 randomly selected individuals aged 20 to 79 years were invited to participate in a comprehensive health examination [29]. A total of 4420 participants (response: 50.1%) gave informed written consent. The study was approved by the ethics committee of the University of Greifswald (ethics approval number BB 39/08) and complies with the Declaration of Helsinki. The study design has been published elsewhere [30].

**Figure 1:**
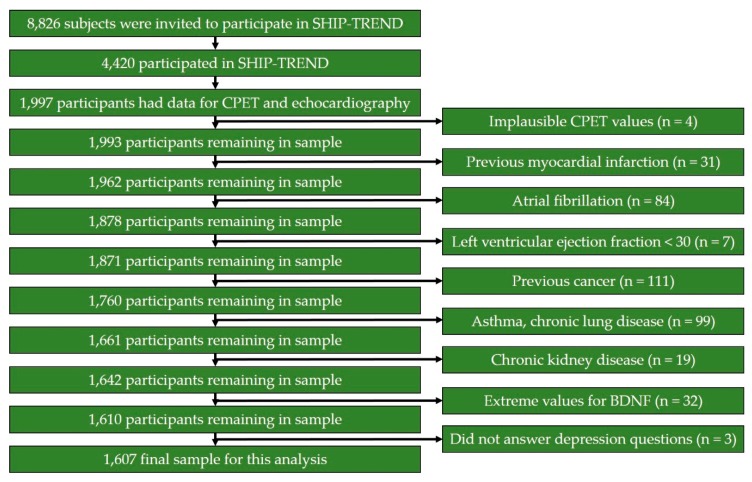
Flow chart of the analysis sample for this investigation was derived.

Individuals with missing cardiopulmonary exercise testing (CPET) or echo values (*n* = 2423), implausible CPET values (*n* = 4), previous myocardial infarction (MI) (*n* = 31), atrial fibrillation (*n* = 84), left ventricular ejection fraction < 30% (*n* = 7), cancer (*n* = 111), chronic lung disease or bronchial asthma (*n* = 99), severe renal (estimated glomerular filtration rate (eGFR) < 30 mL/min/mm^2^) disease (*n* = 19), extreme values for BDNF (*n* = 32) (< 1st and > 99th percentile), or not answering questions with regard to depression (*n* = 3) were excluded (Figure 1). Data from 1607 subjects (785 men and 822 women) were used. The median age was 48 years (25th percentile: 39; 75th percentile: 59 years).

### 2.2. Interview, Medical, and Laboratory Examination

Age, sex, medical history, smoking status, menopause status, and physical inactivity were assessed by computer-assisted personal interviews. Smoking status was classified either as current smoker or non-smoker. Study participants were asked whether they exercised more than one hour per week in the summer or winter. Based on their response, physical inactivity was defined as exercising less than one hour per week during summer and winter. Body mass index (BMI) was calculated by dividing height (m) by weight (kg) squared. Bioelectrical impedance analysis (BIA) was used to measure lean mass and body fat (Nutriguard M, Data Input GmbH, Darmstadt, Germany).

Major depressive disorder (MDD) and recurrent MDD were diagnosed according to DSM-IV using the Munich-Composite International Diagnostic Interview (M-CIDI). The screening questions for depressive disorders comprised the following two items: “Feelings of sadness or depressed mood for a period of at least 2 weeks” and “Lack of interest, tiredness, or loss of energy for a period of at least 2 weeks”.

Diabetes mellitus was defined as a glycosylated hemoglobin A1c level > 6.5%, antidiabetic medication (anatomic, therapeutic, and chemical [ATC] code: A10), or as self-reported based on the question of whether a physician had ever diagnosed diabetes mellitus. Systolic and diastolic blood pressures were assessed after a resting period of 5 min in a sitting position on the right arm. With three minutes rest in between, the blood pressure measurements were taken three times. The average of the second and third measurements is reported. Trained and certified staff used a digital blood pressure monitor (HEM-750CP, Omron Corporation, Tokyo, Japan). Hypertensive patients were identified by either self-reported antihypertensive medication (ATC: C02, C03, C07, C08, and C09) or a systolic blood pressure above 140 mmHg and/or a diastolic value of more than 90 mmHg.

Two-dimensional, M-Mode and Doppler echocardiography were performed using the Vivid-I system (GE Medical Systems, Waukesha, WI, USA) as described in detail elsewhere [31]. Measurement of the left ventricular ejection fraction was performed according to the guidelines of the American Society of Echocardiography [32].

Fasting venous blood samples were collected. Serum samples were subsequently stored at −80 °C. In SHIP-Trend, single-occasion blood samples were drawn from the cubital vein of participants in the supine position following standardized procedures. The sampling was performed between 7:30 and 13:00. The majority (61.2%) of the study participants provided fasting (>8 h) blood samples, and the remaining samples (38.8%) were obtained from non-fasting subjects. A maximum of 65.5 mL of blood was collected in 13 tubes, including EDTA, citrate, serum, and PAXgene tubes. Directly after sampling, EDTA and serum tubes were cooled down to 4 °C, while citrate tubes were stored at room temperature. Hourly transport to the central laboratory (Institute of Clinical Chemistry and Laboratory Medicine, University Medicine Greifswald) was arranged. After arrival at the laboratory, the samples were immediately processed. When necessary, samples were centrifuged at 2550× *g* for 15 min at 8 °C. Then, the samples were analyzed or stored at −80 °C in the Integrated Research Biobank (LiCONiC, Lichtenstein). BDNF levels were measured in serum with a quantitative sandwich enzyme immunoassay technique (Quantikine Human Free BDNF Immunoassay, R&D Systems, Inc., Abington, Science Park, UK). Two concentrations of control material were measured. The coefficients of variation for BDNF were 14.95% at low levels (129 pg/mL) and 5.81% at high levels (667 pg/mL) of control material. The estimated eGFR was calculated: eGFR = 186 × (plasma creatinine concentration × 0.0113118) − 1.154 × age − 0.203; multiplied by 0.742 for female subjects [mL/min/1.73 m^2^] [33]. Serum levels of total cholesterol, low-density lipoprotein (LDL), high-density lipoprotein (HDL), and triglycerides were assessed photometrically (Hitachi 704, Roche, Mannheim, Germany).

### 2.3. Exercise Testing

Cardiopulmonary exercise testing (CPET) was conducted with a calibrated electromagnetically braked cycle ergometer (Ergoselect 100, Ergoline, Bitz, Germany) according to a modified Jones protocol [34,35]. After 3 min of rest and 1 min of unloaded cycling (20 Watts) at 60 rpm, the workload was increased in steps of 16 Watts per minute. The test was terminated by the subject due to exhaustion or by the physician due to ECG abnormalities.

### 2.4. Gas Exchange Variables

During CPET, breath-by-breath gas exchanges were measured by using an Oxycon Pro with a Rudolf’s mask (JÄGER/VIASYS Healthcare System, Hoechberg, Germany). The following parameters were assessed: tidal volume (VE), oxygen uptake (VO_2_), and carbon dioxide uptake (VCO_2_). Furthermore, CPET is coupled continuously with pulse oximetry, blood pressure, and electrocardiogram. The maximal oxygen consumption (VO_2_peak) was defined as the highest 10 s average of VO_2_ during late exercise or early recovery. Oxygen consumption at the aerobic threshold (VO_2_@AT) was detect by the nonlinear increase of VE in relation to VO_2_, as described in Wassermann et al. [36]. The VE/VCO_2_ slope demonstrates the relation between VE (*y*-axis) and VCO_2_ (x-Axis). Peak oxygen pulse (O2HRmax) was defined as VO_2_peak divided by maximal heart rate. The participants had to have a respiratory exchange ratio greater than 1.1, blood lactate levels higher than 8 mmol/L, or a BORG rating of perceived exhaustion larger/equal 18.

### 2.5. Statistics

The normality and homoscedasticity of residuals were assessed using histograms, kernel density plots, Q-Q plots, and residuals-vs-fitted plots. Sex and age-specific VO_2_peak quartiles are used to describe the study population. For descriptive statistics, we created age and sex-specific quartiles. We first assessed the interaction between the CPET parameters and sex by including the interaction term in the fully adjusted multivariable regression mode. Thereafter, sex-specific linear regression models were used to relate CRF parameters and serum BDNF. All models were adjusted for age, smoking, body fat, lean mass, depression, and physical inactivity. Previous research suggested that peripheral BDNF levels are largely determined by platelet activation [37]. Hence, in a third step, platelets were added as a potential confounder. Potential nonlinear associations were tested with restricted cubic splines. Three knots were pre-specified, located at the 5th, 50th, and 95th percentiles [38], resulting in one component of the spline function. Variance inflation factor (VIF) analysis was used to assess the potential multicollinearity among the confounders. All the calculations were done in SAS 9.4 (SAS Institute, Cary, NC, USA). Statistical significance was defined as *p* < 0.05.

## 3. Results

### 3.1. General Characteristics

The population description according to VO_2_peak quartiles for men and women are presented in Table 1 and Table 2, respectively. There were no significant differences between VO_2_peak quartiles with regard to hypertension, left ventricular ejection fraction, eGFR, and diabetes mellitus. In males, the VO_2_peak quartiles were significantly different considering the prevalence of BMI, smoking, physical inactivity, major depressive disorders, lean mass, and HDL cholesterol. In females, differences between quartiles were found for BMI, smoking, physical inactivity, fat mass, lean mass, and HDL cholesterol.

### 3.2. The Association between BDNF and CRF

**Figure 2:**
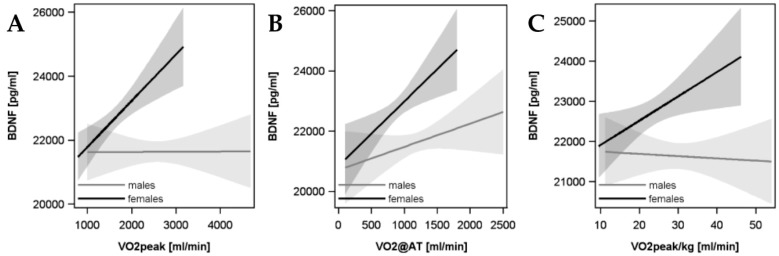
Association between VO_2_peak (**A**), VO_2_@AT (**B**), and VO_2_peak/kg (**C**) with circulating BDNF levels. BDNF, brain-derived neurotropic factor; VO_2_peak, maximal oxygen consumption; VO_2_peak/kg, maximal oxygen consumption adjusted for body weight; VO_2_@AT, maximal oxygen consumption at the anaerobic threshold.

Figures illustrating that the assumptions for using multivariable linear regression models were met are presented in the Supplementary Materials, Figures S1–S12. Specifically, the homoscedasticity of the residuals is shown in residuals-vs-fitted plots (top left caption). Plots that show the normal distribution of the residuals are shown in the Q-Q plots (middle left caption). The variance inflation factors for all parameters in the model are shown in the Supplementary Materials, Table 1 and Table 2. We identified significant interactions between VO_2_peak and sex with regard to the association with BDNF (β coefficient 1.00 standard error 0.57; *p* = 0.08). CRF was significantly positively associated with circulating BDNF serum levels in women, but not men (Figure 2). Specifically, in women, a 1 mL/min higher VO_2_peak was associated with a 2.43 pg/mL (95% CI: 1.16 to 3.69 pg/mL; *p* = 0.0002, R^2^ = 0.0367) greater BDNF concentration. A 1 mL/min/kg higher VO_2_peak/kg was related with a 150.66 pg/mL (95% CI: 63.42 to 237.90 pg/mL; *p* = 0.0007, R^2^ = 0.0335) larger BDNF concentration. A 1 mL/min higher VO_2_@AT was associated with an increase of 2.68 pg/mL (95% CI: 0.53 to 4.82 pg/mL; *p* = 0.015, R^2^ = 0.0269) of BDNF.

In men, a 1 mL/min greater VO_2_peak (β = 0.43; 95% CI: −0.46 to 1.31 pg/mL; *p* = 0.35, R^2^ = 0.0166) and VO_2_@AT (β = 1.41; 95% CI: −0.20 to 3.02 pg/mL; *p* = 0.09, R^2^ = 0.0194) was not significantly associated with serum BDNF levels. Further, a 1 mL/min/kg increase in VO_2_peak/kg (β = 18.88; 95% CI: −56.44 to 94.19 pg/mL; *p* = 0.62, R^2^ = 0.0158) was also not related to peripheral BDNF.

We performed a stratified analysis for women before and after menopause, respectively. Before and after menopause, a 1 mL/min increase in VO_2_peak was associated with a BDNF increase of 2.07 pg/mL (95% CI: 0.46 to 3.68 pg/mL; *p* = 0.01) and 2.47 pg/mL (95% CI: 0.41 to 4.53 pg/mL; *p* = 0.02), respectively. Further, we assessed whether the differences before and after menopause were different by including the interaction for VO_2_peak and menopause into the model. This was not statistically significant (*p* = 0.34).

Additional adjustment for platelet count did not significantly influence our results. In this analysis, a 1 mL/min higher VO_2_peak was associated with a 2.35 pg/mL greater BDNF level (95% CI: 1.17 to 3.52 pg/mL; *p* < 0.01, R^2^ = 0.1755) in women, but not in men (β = 0.53; 95% CI: −0.28 to 1.35 pg/mL; *p* = 0.20, R^2^ = 0.1649). In men, the association between VO_2_@AT and BDNF levels became significant after adjustment for platelet count. VO_2_@AT was positively associated with BDNF in men (β = 1.69; 95% CI: 0.21 to 3.18 pg/mL; *p* = 0.03, R^2^ = 0.1709) and women (β = 2.85; 95% CI: 0.87 to 4.84 pg/mL; *p* = 0.01, R^2^ = 0.1709).

## 4. Discussion

This investigation explored the relation between CRF and peripheral serum levels of BDNF. Previous studies demonstrated that acute aerobic exercise and long-term training programs increase peripheral BDNF levels [12,13,14,39,40]. However, very few studies have examined whether cardiorespiratory exercise capacity is related to BDNF. Here, we report that greater values of peak oxygen uptake, peak oxygen uptake adjusted for body weight, and oxygen uptake at the anaerobic threshold are associated with higher BDNF serum levels in women, but not men. Further, we report that this observation was independent of menopause status in women.

A meta-analysis included data of 32 publications investigating the relation between physical activity and exercise with circulating BDNF in healthy humans [41]. This analysis included nine observational studies and 15 as well as six studies that explored the relation between acute and chronic exercise on peripheral BDNF levels, respectively. Of the nine observational investigations, five reported an inverse relationship between BDNF and habitual physical activity or cardiorespiratory fitness. A total of 14 out of the 15 experimental studies demonstrated that BDNF concentrations increased in response to acute aerobic exercise, but returned to baseline immediately after. Four out of six studies reported that chronic endurance exercise training increased resting BDNF. Hence, aerobic exercise training that increased cardiorespiratory exercise capacity was associated with elevated BDNF. Thus, one may speculate that higher levels of CRF are also related with greater BDNF. This hypothesis is supported by our findings.

To our knowledge, very few studies investigated the relation between CRF and BDNF [16,18,19,42]. In contrast to our findings, the majority of these studies reported inverse associations [16,18,19]. However, merely two studies included both sexes [16,42]. Specifically, in a small cross-sectional sample (*n* = 44) of subjects without overt cardiometabolic disease, BDNF concentrations decreased with increasing levels of physical activity and higher estimated VO_2_peak [16]. In contrast, a second cross-sectional study (*n* = 88) reported a positive association between CRF and BDNF in older, largely sedentary patients with coronary artery disease (mean age: 63 years, 85% male) [42]. The small sample sizes in these studies did not allow sex-specific analyses. In our study of 822 women and 785 men, a positive association between CRF and BDNF was only shown for women.

There are several reasons that might explain why our results differ from some of the previous reports. The heterogeneous findings may be related to differences regarding the study populations. For example, earlier studies were based on smaller sample sizes [16], and included individuals without overt cardiometabolic disease [16,18,19]. In contrast, we were able to use data from the population-based SHIP, which included individuals with, for example, metabolic (6.4% diabetes mellitus) and cardiovascular diseases (38.7% hypertension). Moreover, previous reports regarding the association between physical activity and BDNF were based on studies that included mostly males. Very few studies included both sexes [12,13,15]. Thus, there was a relevant need to investigate whether the results obtained from males are also applicable to women. Our results suggest that this is not the case. The different findings of our study compared to previous investigations may also be related to the consideration of confounders. While some studies did not make any adjustments at all [16,19], one study adjusted for age, BMI, triglycerides (TC), and the ratio TC/HDL [18], and another one adjusted for depression, age, sex, val66met genotype, and serum inflammatory markers [42]. In contrast, we used a multivariable approach adjusting for several confounders (age, height, lean mass, fat mass, depression, smoking, and physical activity). In addition, the definition of physical activity differs in many publications. Currie et al. [16] used a modified Baecke questionnaire, while Nofuji et al. [17] used a Lifecorder for one week to calculate the basal metabolic consumption, daily total energy expenditure, and movement-related energy expenditure. We simply defined physically inactive individuals based on less than one hour of exercise per week. This may have influenced the findings, and could partly explain the different results.

A biological hypothesis explaining why we observed significant associations in women only may be related to estrogen (E2). A hypothetical model recently linked E2 and BDNF with peroxisome proliferator-activated receptor gamma coactivator 1-alpha (PGC1α) synthesis to improve mitochondrial function [43]. However, in our stratified analysis, higher BDNF levels were related with greater VO_2_peak values independent of menopause. Thus, our results do not support the conclusion that the observed sex-specific differences are driven by E2. However, the BDNF related differences between men and women may be attributable to sex-specific skeletal muscle composition (i.e., more type I fibers in women) [44]. Whereas, to the best of our knowledge, no published data are available concerning variations of BDNF secretion by muscle fiber type, one may speculate that type 1 fibers secrete more BDNF, since this neurotrophin increases fat oxidation in human C2C12 skeletal muscle cells [11]. Type 1, but not type 2 fibers, mainly utilize fat as an energy substrate. Interestingly, BDNF was recently identified to regulate and promote a glycolytic muscle fiber phenotype in young male mice [27]. However, in female mice, a lack of BDNF resulted in skeletal muscle metabolic myopathy and insulin resistance [28]. Further research is required to confirm and fully understand potential sex-specific associations and their underlying mechanisms between cardiorespiratory fitness, skeletal muscle function, and BDNF.

Regarding the association of CRF and BDNF levels, another point need to be stressed. Rather than stating that an increase in CRF is related with a higher BDNF level, the results could also be inferred the other way around. Then, in women, a 1 mL/min less VO_2_peak would be related to 2.43 pg/mL lower BDNF. Since our study population has a very broad age range, we may interpret our findings in the context of cross-sectional aging. SHIP participants were between 20 and 79 years old. During aging, over 60 years, the human body loses around 30% of its skeletal muscle mass [45], and cardiorespiratory fitness decreases by approximately 40% [46]. Further, the number of mitochondria in skeletal muscle as well as their enzymatic content is reduced [47]. BDNF has been proposed to play an essential function in the regulation of mitochondrial function [48]. Hence, aging is not only related to a decrease in CRF, but also in loss of muscle mass as an important secretion site of BDNF. This may explain why lower CRF is associated with less BDNF (released from less skeletal muscle with advanced age). Despite the fact that we adjusted for age, our observations may just be a surrogate for the aging muscle throughout life. Even though we used BIA measurements to adjust for body composition, we cannot exclude the possibility that sex-specific skeletal muscle atrophy and sarcopenia may have had an impact of our results [49,50].

The large number of individuals (*n* = 1607) and the use of standardized data collection methods for our analyses are the strengths of this study. However, the results of this study need to be interpreted in the context of some limitations. Our results are not directly applicable to other ethnicities, because SHIP consists of Caucasians. Another limitation is that we are unable to ascertain whether changes in BDNF are also associated with alterations in cardiorespiratory fitness due to the cross-sectional nature of available data. We acknowledge that our approach for assessing physical inactivity has limits, and probably underestimates the true number of individuals with a sedentary lifestyle. In addition, the ELISA used to measure BDNF does not differentiate between mature BDNF and its precursor proBDNF. Both isoforms have opposite effects via TrkB, and are involved in several physiological functions [51]. Even though their exact functions are yet not known, we cannot exclude the possibility that higher CRF shifts the ratio between mature and proBDNF. Future research needs to assess the association between the different BDNF isoforms with CRF. Lastly, while we used a directed acyclic graph to identify potential confounders, we cannot exclude the possibility of additional residual confounding that may have influenced the results of our analysis.

## 5. Conclusions

This study is the first to show that higher levels of circulating BDNF are associated with greater cardiorespiratory fitness in women, but not men.

## Figures and Tables

**Table 1:** Population descriptions according to VO_2_peak quartiles for males.

		VO_2_peak Quartiles				
		I	II	III	IV	
	mL/min	1667–2155	2104–2606	2409.5–2953	2822–3454	*p* for Trend
n		191	199	196	199	
Age (years)		49 (39; 59)	49 (39; 59)	49 (39; 58)	49 (38; 59)	0.9167
BDNF (ng/mL)		21.76 (17.33; 25.86)	21.73 (17.23; 26.77)	20.87 (17.34; 24.58)	21.50 (17.96; 25.63)	0.4102
Risk factors	BMI (kg/m^2^)	27.0 (24.4; 29.5)	27.1 (25.0; 29.6)	27.6 (25.5; 30.7)	27.9 (25.4; 30.4)	**0.0236**
	Hypertension (%)	50	47.47	50.77	41.21	0.2155
	Systolic BP (mmHg)	132 (120; 143)	132 (125; 142)	133 (123; 142)	132 (122; 142)	0.8439
	Diabetes mellitus (%)	7.9	7.5	7.7	4	0.3654
	LVEF (%)	69 (63; 77)	71 (66; 77)	72 (65; 79)	71 (65; 77)	0.2617
	Smoking (%)	42.4	25.3	22	10.6	**<0.0001**
	Physical inactivity (%)	31.4	19.1	16.8	10	**<0.0001**
	eGFR (mL/min/1.72 mm^2^)	103 (89; 115)	106 (96; 115)	106 (97; 114)	106 (95; 113)	0.3049
BIA	Fat mass (%)	23.2 (19.2; 26.8)	23.65 (19.6; 26.5)	23.7 (19.6; 27)	22.45 (19; 25.4)	0.1457
	Lean mass (kg)	63.3 (57.9; 68.3)	64.7 (60; 69.9)	67.4 (62.8; 72.7)	69.65 (64.9; 74.4)	**<0.0001**
	ECM (kg)	29.2 (26.3; 31.9)	28.9 (27.2; 31.6)	30.1 (27.7; 33.2)	30.9 (28.5; 34.0)	**<0.0001**
	BCM (kg)	34.3 (30.2; 37.6)	35.2 (32.8; 38.6)	37.0 (34.3; 40.0)	38.6 (35.2; 41.6)	**<0.0001**
	BW (L)	46.4 (42.4; 50.0)	47.4 (43.9; 51.2)	49.4 (46.1; 53.2)	50.9 (47.5; 54.5)	**<0.0001**
Lipids	Total cholesterol (mmol/L)	5.5 (4.7; 6.4)	5.3 (4.6; 6.1)	5.4 (4.7; 6.2)	5.3 (4.5; 6)	0.1398
	TG (mmol/L)	1.4 (1.01; 2.37)	1.35 (0.91; 1.98)	1.43 (0.94; 2.16)	1.25 (0.9; 1.89)	0.0573
	LDLC (mmol/L)	3.58 (2.89; 4.19)	3.4 (2.78; 3.98)	3.44 (2.89; 4.0)	3.34 (2.68; 3.92)	0.1653
	HDL Chol (mmol/L)	1.22 (1.02; 1.44)	1.3 (1.11; 1.53)	1.25 (1.09; 1.47)	1.33 (1.14; 1.55)	**0.0016**
Depression	Feelings of sadness/depressed mood for a period of at least 2 weeks (%)	39.27	32.83	29.08	34.67	0.2003
	Lack of interest, tiredness, or loss of energy for a period of at least 2 weeks (%)	23.04	20.71	11.22	9.55	**0.0002**
CPET	VO_2_ peak (mL/min/kg)	22.4 (19.0; 26.6)	27.8 (24.6; 31.6)	29.9 (26.2; 35.7)	34.5 (30.3; 41.2)	**<0.0001**
	VO_2_AT (mL/min)	900 (850; 1050)	1100 (950; 1250)	1200 (1100; 1350)	1400 (1250; 1550)	**<0.0001**
	Watt max	164 (132; 180)	196 (164; 228)	212 (180; 244)	244 (212; 276)	**<0.0001**
	HR max (/min)	153 (137; 171)	166 (148; 181)	166 (148; 179)	171 (162; 181)	**<0.0001**

Values presented as median (25th and 75th percentile). For categorical variables, percentage is provided. BDNF, brain-derived neurotrophic factor; BMI, body mass index; systolic BP, systolic blood pressure; eGFR, estimated glomerular filtration rate; ECM, extracellular mass; BCM, body cell mass; BW, body water; CPET, cardiopulmonary exercise testing; TG, triglycerides; HDLC, high-density lipoprotein cholesterol; LDLC, low-density lipoprotein cholesterol; VO_2_peak, maximal oxygen consumption; VO_2_@AT, maximal oxygen consumption at the anaerobic threshold; HR max, maximal heart rate; LVEF, left ventricular ejection fraction; BIA, body impedance analysis. Bold lettering of the p-value indicates a significance for trend (*p* < 0.05).

**Table 2:** Population descriptions according to VO_2_peak quartiles for females.

		VO_2_peak Quartiles				
		I	II	III	IV	
	mL/min	1118.5–1370	1391–1650	1566–1900	1885–2200	*p* for Trend
n		204	200	204	214	
Age (years)		48 (38; 59)	47 (38; 58)	48 (38; 59)	47 (39; 60)	0.9883
BDNF (ng/mL)		22.09 (18.58; 26.30)	22.65 (18.34; 25.87)	22.3 (18.68; 26.88)	23.34 (19.38; 27.23)	0.1427
Risk factors	BMI (kg/m^2^)	24.4 (21.8; 28.0)	25.5 (23.2; 27.9)	26.3 (23.4; 30.1)	26.9 (24.0; 30.5)	**<0.0001**
	Hypertension (%)	31.4	27.6	31.9	31.3	0.7811
	Systolic BP (mmHg)	116 (107; 129)	117 (108; 130)	116 (106; 127)	118 (111; 128)	0.1902
	Diabetes mellitus (%)	4.9	8	7.4	3.7	0.2139
	LVEF (%)	72 (67; 77)	73 (67; 79)	73 (67; 78)	75 (67; 80)	0.5086
	Smoking (%)	31.4	24.5	17.7	18.2	**0.0211**
	Physical inactivity (%)	25	16.5	13.7	7.5	**<0.0001**
	eGFR (mL/min/1.72 mm^2^)	106 (92; 116)	105 (93; 114)	104 (91; 113)	104 (93; 114)	0.2082
BIA	Fat mass (kg)	31.75 (26.8; 36.6)	32.6 (28.2; 36.4)	33.75 (28.75; 38.65)	34.4 (28.9; 38.2)	**0.0084**
	Lean mass (kg)	44.45 (41.2; 47.8)	46.4 (43.6; 48.9)	47.3 (44.4; 51.6)	49.4 (46.7; 52.4)	**<0.0001**
	ECM (kg)	21.9 (20.1; 23.5)	22.5 (20.9; 24.3)	23.1 (21.5; 25.1)	23.5 (22.0; 25.5)	**< 0,0001**
	BCM (kg)	22.5 (20.7; 24.6)	23.6 (21.9; 25.2)	24.6 (22.5; 26.8)	25.7 (24.0; 27.9)	**<0.0001**
	BW (L)	32.5 (30.2; 35.0)	34.0 (32.0; 35.8)	34.7 (32.6; 37.8)	36.1 (34.2; 38.4)	**<0.0001**
Lipids	Total cholesterol (mmol/L)	5.5 (4.9; 6.3)	5.5 (4.8; 6.2)	5.2 (4.6; 6.0)	5.5 (4.8; 6.3)	0.0467
	TG (mmol/L)	1.2 (0.9; 1.8)	1.1 (0.8; 1.6)	1.2 (0.9; 1.6)	1.1 (0.8; 1.5)	0.1023
	LDLC (mmol/L)	3.3 (2.8; 4.0)	3.3 (2.7; 4.1)	3.2 (2.6; 3.8)	3.3 (2.7; 3.9)	0.6075
	HDL Chol (mmol/L)	1.6 (1.4; 1.9)	1.6 (1.4; 1.9)	1.6 (1.3; 1.8)	1.6 (1.4; 1.8)	**0.2558**
Depression	Feelings of sadness/depressed mood for a period of at least 2 weeks (%)	53.43	54.5	51.93	49.07	0.7066
	Lack of interest, tiredness, or loss of energy for a period of at least 2 weeks (%)	39.22	37.5	38.73	32.71	**0.4934**
CPET	VO_2_ peak (mL/min/kg)	18.6 (16.0; 22.0)	22.4 (19.4; 25.2)	23.7 (19.8; 28.3)	26.9 (23.1; 31.8)	**<0.0001**
	VO_2_AT (mL/min)	700 (650; 800)	800 (750; 900)	900 (800; 975)	1050 (900; 1150)	**<0.0001**
	Watt max	116 (84; 132)	132 (116; 148)	148 (116; 148)	164 (148; 180)	**<0.0001**
	HR max (/min)	156 (134; 169)	160 (142; 173)	164 (148; 173)	166 (151; 176)	**<0.0001**

Values presented as median (25th and 75th percentile). For categorical variables, percentage is provided. BMI, body mass index; systolic BP, systolic blood pressure; eGFR, estimated glomerular filtration rate; ECM, extracellular mass; BCM, body cell mass; BW, body water; TG, triglycerides; HDLC, high-density lipoprotein cholesterol; LDLC, low-density lipoprotein cholesterol; VO_2_peak, maximal oxygen consumption; VO_2_@AT, maximal oxygen consumption at the anaerobic threshold; HR max, maximal heart rate; LVEF, left ventricular ejection fraction; BIA, body impedance analysis. Bold lettering of the p-value indicates a significance for trend (*p* < 0.05).

## Supplementary Materials

The following are available online at https://www.mdpi.com/2218-273X/9/10/630/s1, Table S1: Variance inflation factors for the different model, Figure S1: BDNF distribution in males and females, Figure S2: Fit diagnostics for the association between BDNF and VO_2_peak in males, Figure S3: Fit diagnostics for the association between BDNF and VO_2_peak in females, Figure S4: Fit diagnostics for the association between BDNF and VO_2_peak/kg in males, Figure S5: Fit diagnostics for the association between BDNF and VO_2_peak/kg in females, Figure S6: Fit diagnostics for the association between BDNF and VO_2_@AT in males, Figure S7: Fit diagnostics for the association between BDNF and VO_2_@AT in females, Figure S8: Fit diagnostics for the association between BDNF and VO_2_peak in males with additional adjustment for platelets, Figure S9: Fit diagnostics for the association between BDNF and VO_2_peak in females with additional adjustment for platelets, Figure S10: Fit diagnostics for the association between BDNF and VO_2_peak/kg in males with additional adjustment for platelets, Figure S11: Fit diagnostics for the association between BDNF and VO_2_peak/kg in females with additional adjustment for platelets, Figure S12: Fit diagnostics for the association between BDNF and VO_2_@AT in males with additional adjustment for platelets, Figure S13: Fit diagnostics for the association between BDNF and VO_2_@AT in females with additional adjustment for platelets.
