# Supplementary material for: Sex-Specific Associations of Brain-Derived Neurotrophic Factor and Cardiorespiratory Fitness in the General Population
Source: Biomolecules. 2019 Oct 20;9(10):630. doi: 10.3390/biom9100630 (PMC6843272; doi:10.3390/biom9100630)
Supplement: Supplementary file 1 [file biomolecules-09-00630-s001.pdf]

Supplementary material for

Sex-specific associations of brain-derived neurotrophic factor and cardiorespiratory fitness in the general population by Schmalhofer et al.

Content:

Suppl. Table 1 – Variance inflation factors for the different model
Suppl. Figure 1 – BDNF distribution in males and females
Suppl. Figure 2 – Fit diagnostics for the association between BDNF and VO_2peak in males
Suppl. Figure 3 – Fit diagnostics for the association between BDNF and VO_2peak in females
Suppl. Figure 4 – Fit diagnostics for the association between BDNF and VO_2peak/kg in males
Suppl. Figure 5 – Fit diagnostics for the association between BDNF and VO_2peak/kg in females
Suppl. Figure 6 – Fit diagnostics for the association between BDNF and VO_2@AT in males
Suppl. Figure 7 – Fit diagnostics for the association between BDNF and VO_2@AT in females
Suppl. Figure 8 – Fit diagnostics for the association between BDNF and VO_2peak in males with additional adjustment for platelets
Suppl. Figure 9 – Fit diagnostics for the association between BDNF and VO_2peak in females with additional adjustment for platelets
Suppl. Figure 10 – Fit diagnostics for the association between BDNF and VO_2peak/kg in males with additional adjustment for platelets
Suppl. Figure 11 – Fit diagnostics for the association between BDNF and VO_2peak/kg in females with additional adjustment for platelets
Suppl. Figure 12 – Fit diagnostics for the association between BDNF and VO_2@AT in males with additional adjustment for platelets
Suppl. Figure 13 – Fit diagnostics for the association between BDNF and VO_2@AT in females with additional adjustment for platelets

Variable	DF	Males			Females		
		VIF for VO_2peak	VIF for VO_2peak/kg	VIF for VO_2@AT	VIF for VO_2peak	VIF for VO_2peak/kg	VIF for VO_2@AT
Intercept	1	0	0	0	0	0	0
Response variable	1	1.84602	2.15087	1.29499	1.63618	1.86427	1.23074
Age	1	1.69128	1.74452	1.24157	1.54406	1.55876	1.29044
Body fat in kg	1	1.51361	1.81096	1.49444	2.07041	2.62105	2.0471
Body lean mass in kg	1	1.68868	1.45687	1.5927	2.32168	1.99691	2.14338
Current smoking	1	1.19257	1.19849	1.14676	1.17619	1.17861	1.18316
Physical inactivity	1	1.07513	1.07611	1.06311	1.07965	1.0777	1.05586
Depression 1	1	1.17758	1.17648	1.15798	1.28807	1.28803	1.28797
Depression 2	1	1.14436	1.14526	1.14208	1.28719	1.28718	1.2872
Platelet count	1				1.06015	1.06011	1.06023

Suppl. Table 1 – Variance inflation factors (VIF) for the different models. VO_2peak – peak oxygen consumption, VO_2peak/kg – peak oxygen consumption normalized to body weight, VO_2@AT – oxygen consumption at the aerobic threshold

Suppl. Figure 1

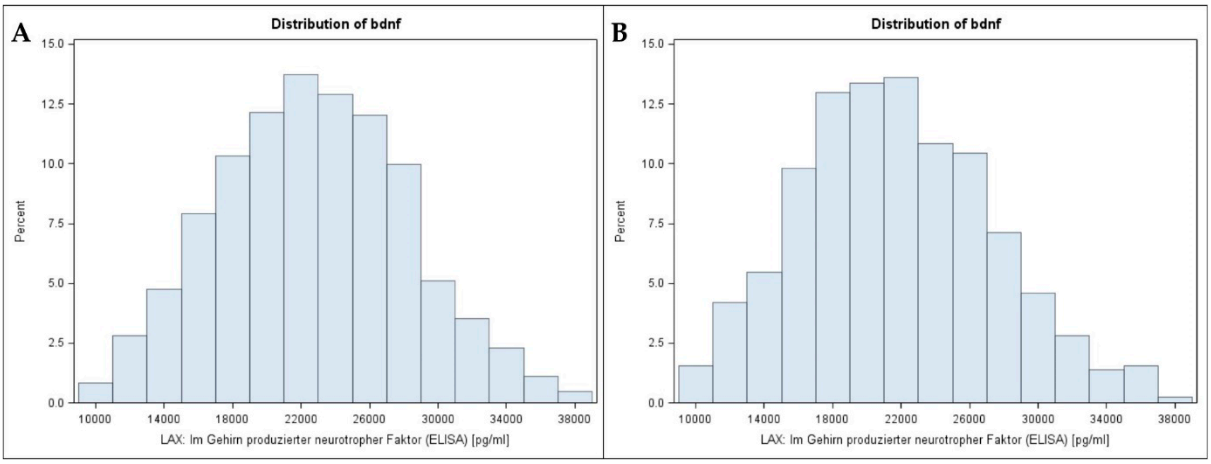

*Suppl. Figure 1 – Histograms show the distribution of BDNF in males (**A**) and females (**B**).*

Suppl. Figure 2 – Fit diagnostics for the association between BDNF and VO_2peak in males.

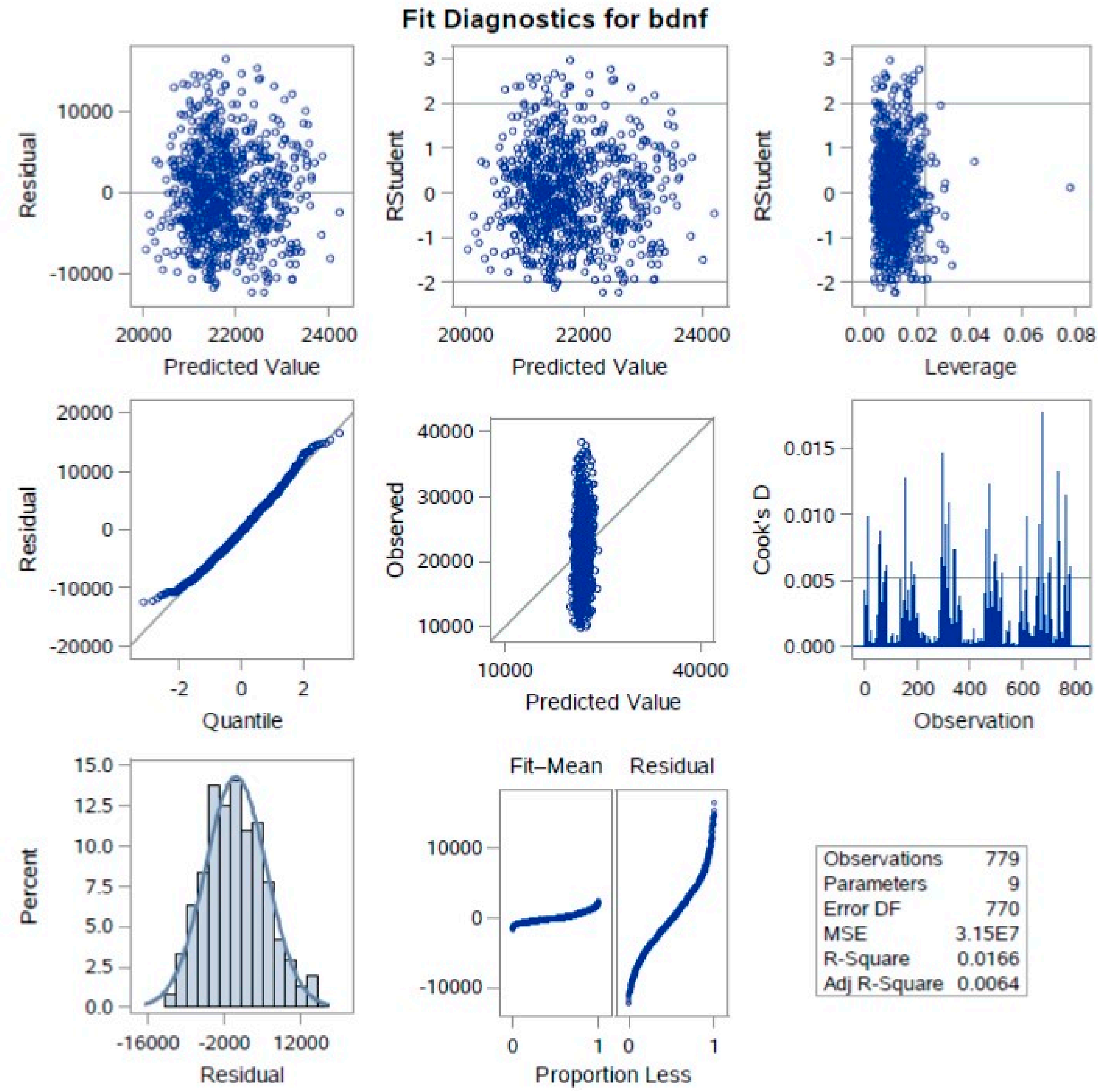

Suppl. Figure 2 – Fit diagnostics for the association between BDNF and VO_2peak in males. Residual vs. predicted value, RStudent vs. predicted value, Rstudent vs. leverage, residual vs. quantile, predicted vs. observed, Cook's D and residual distribution plots.

Suppl. Figure 3 – Fit diagnostics for the association between BDNF and VO_2peak in females.

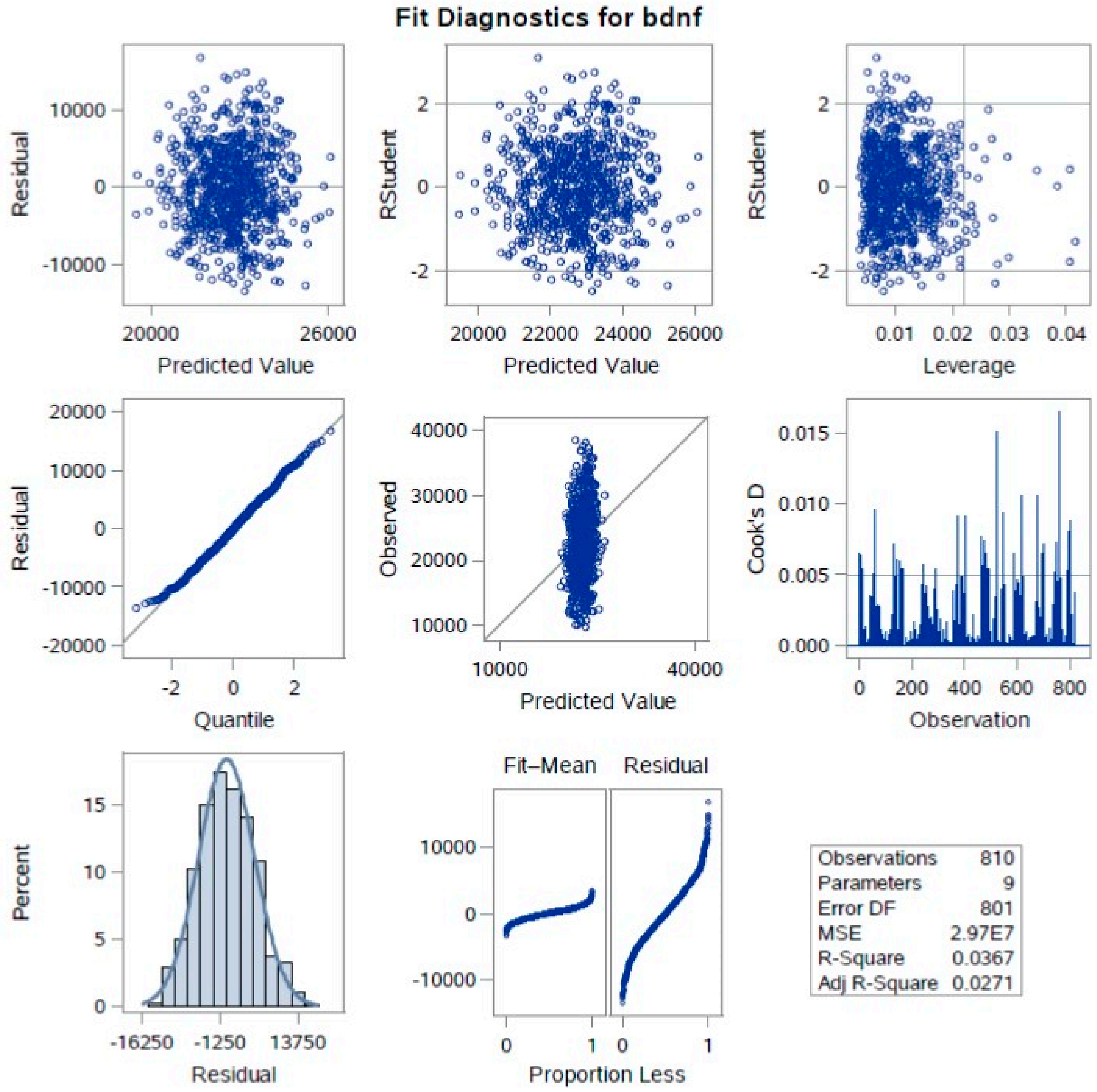

Suppl. Figure 3 – Fit diagnostics for the association between BDNF and VO_2peak in females. Residual vs. predicted value, RStudent vs. predicted value, Rstudent vs. leverage, residual vs. quantile, predicted vs. observed, Cook's D and residual distribution plots.

Suppl. Figure 4 – Fit diagnostics for the association between BDNF and VO_2peak/kg in males.

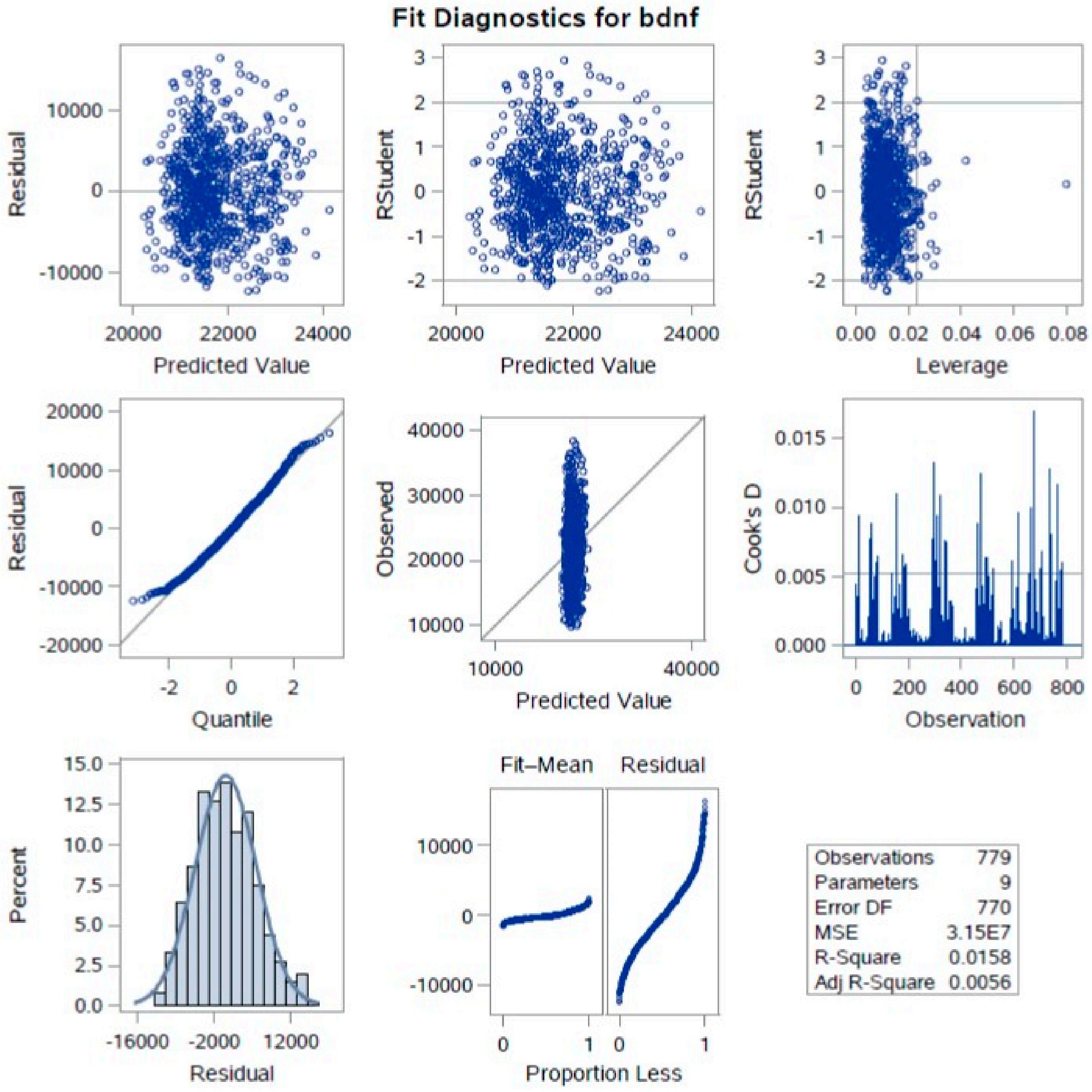

Suppl. Figure 4 – Fit diagnostics for the association between BDNF and VO_2peak/kg in males. Residual vs. predicted value, RStudent vs. predicted value, Rstudent vs. leverage, residual vs. quantile, predicted vs. observed, Cook's D and residual distribution plots.

Suppl. Figure 5 – Fit diagnostics for the association between BDNF and VO_2peak/kg in females.

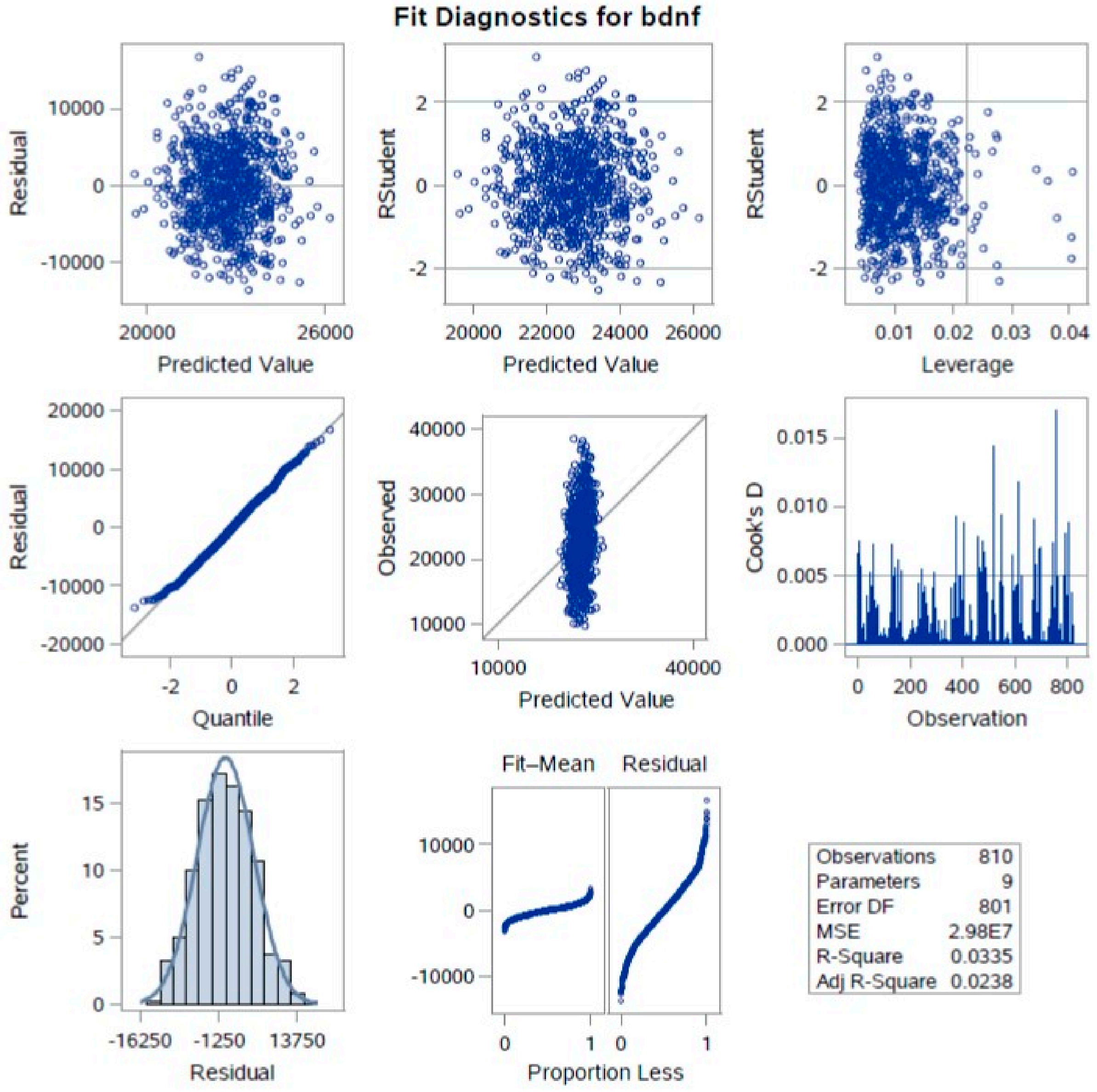

Suppl. Figure 5 – Fit diagnostics for the association between BDNF and VO_2peak/kg in females. Residual vs. predicted value, RStudent vs. predicted value, Rstudent vs. leverage, residual vs. quantile, predicted vs. observed, Cook's D and residual distribution plots.

Suppl. Figure 6 – Fit diagnostics for the association between BDNF and VO_2@AT in males.

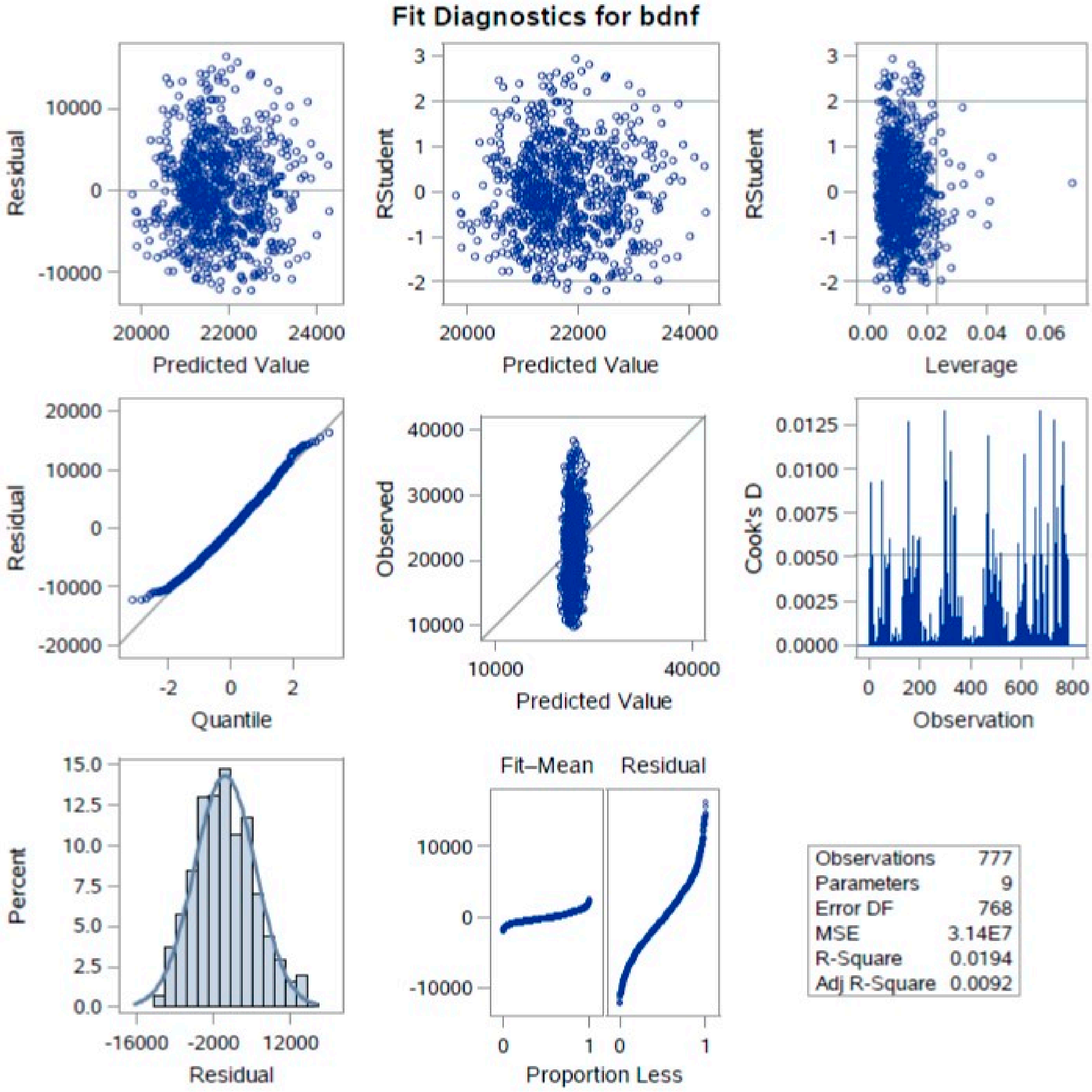

Suppl. Figure 6 – Fit diagnostics for the association between BDNF and VO_2@AT in males. Residual vs. predicted value, RStudent vs. predicted value, Rstudent vs. leverage, residual vs. quantile, predicted vs. observed, Cook's D and residual distribution plots.

Suppl. Figure 7 – Fit diagnostics for the association between BDNF and VO_2@AT in females.

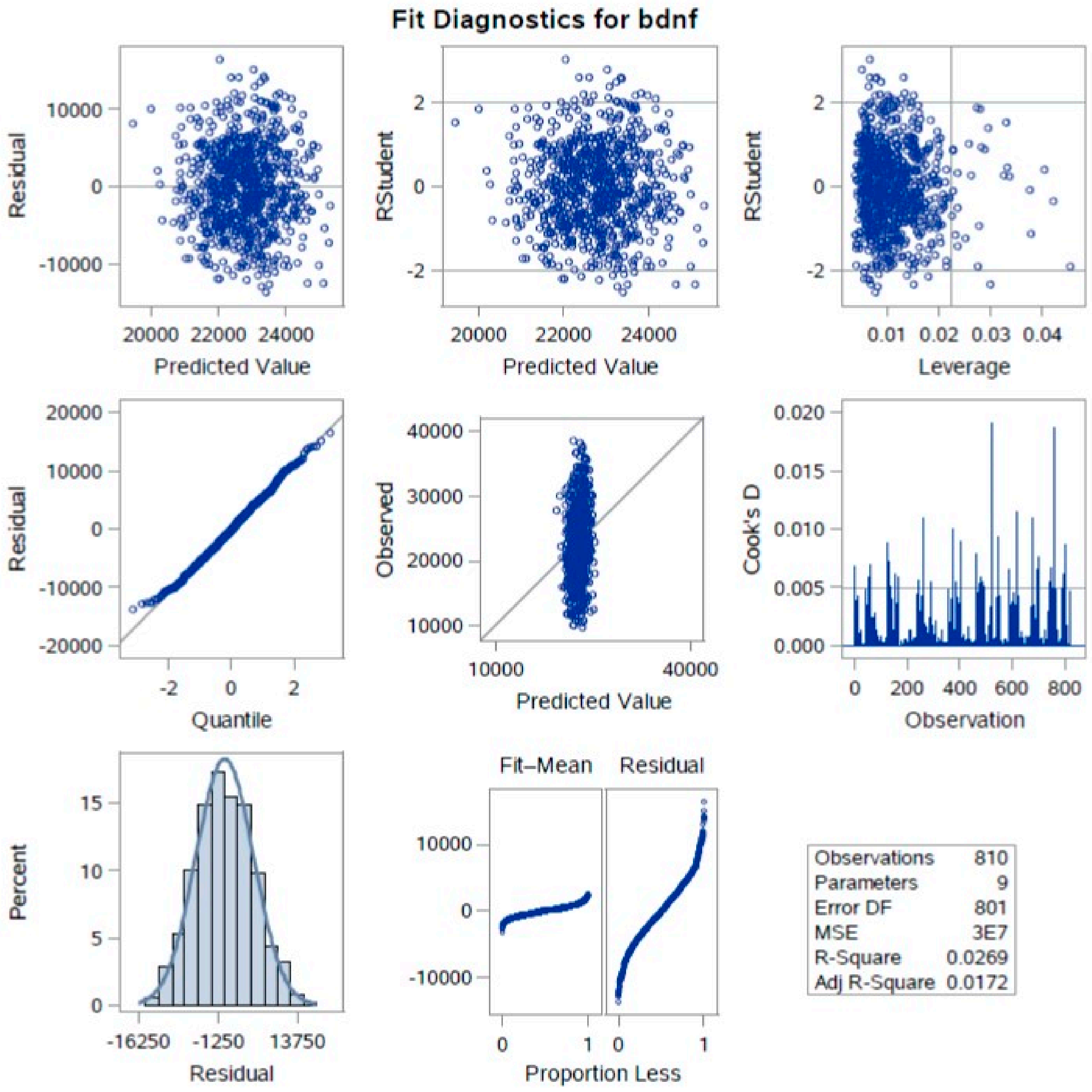

Suppl. Figure 7 – Fit diagnostics for the association between BDNF and VO_2@AT in females. Residual vs. predicted value, RStudent vs. predicted value, Rstudent vs. leverage, residual vs. quantile, predicted vs. observed, Cook's D and residual distribution plots.

Suppl. Figure 8 – Fit diagnostics for the association between BDNF and VO_2peak in males with additional adjustment for platelets.

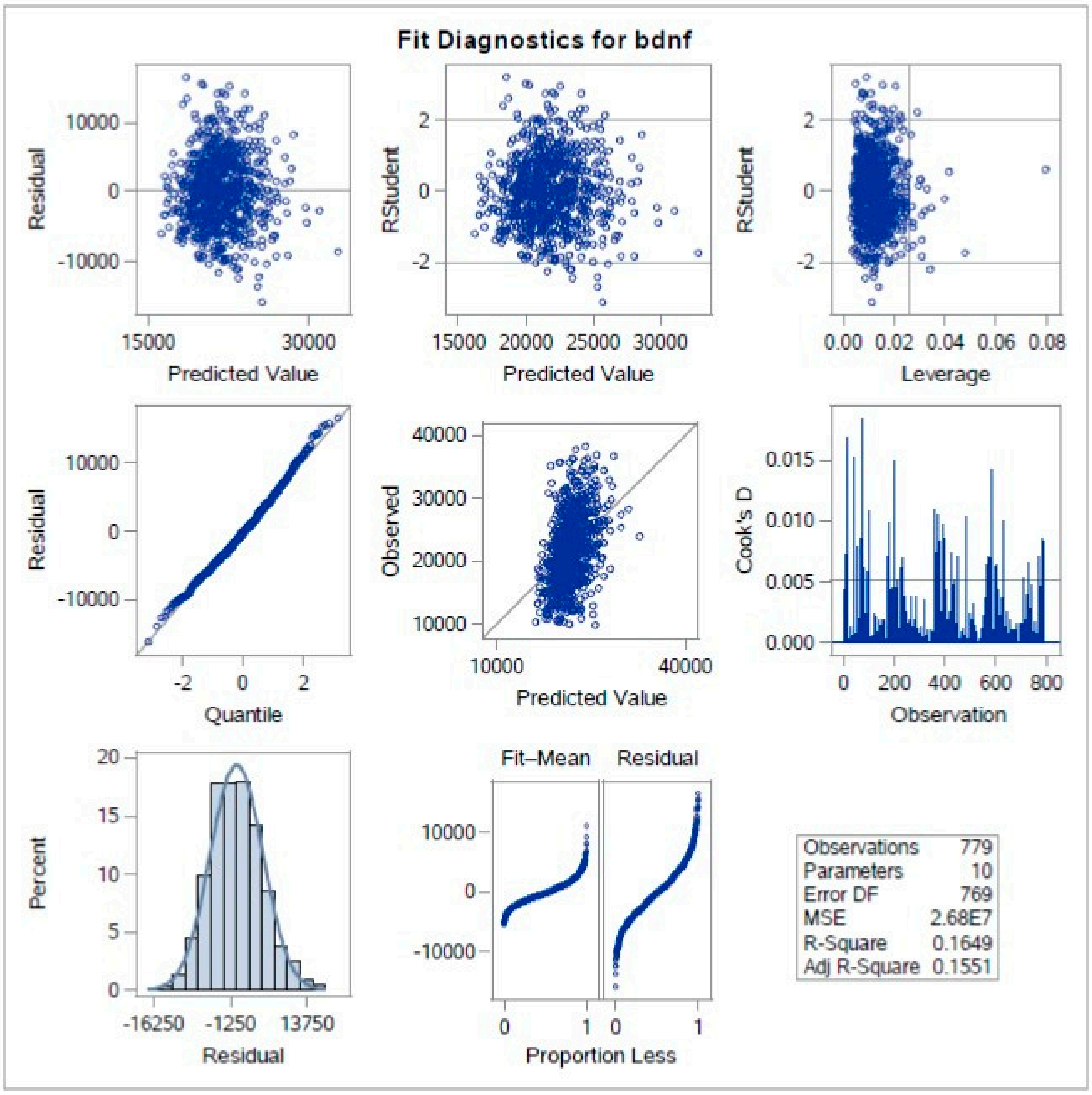

Suppl. Figure 8 – Fit diagnostics for the association between BDNF and VO_2peak in males with additional adjustment for platelets. Residual vs. predicted value, RStudent vs. predicted value, Rstudent vs. leverage, residual vs. quantile, predicted vs. observed, Cook's D and residual distribution plots.

Suppl. Figure 9 – Fit diagnostics for the association between BDNF and VO_2peak in females with additional adjustment for platelets.

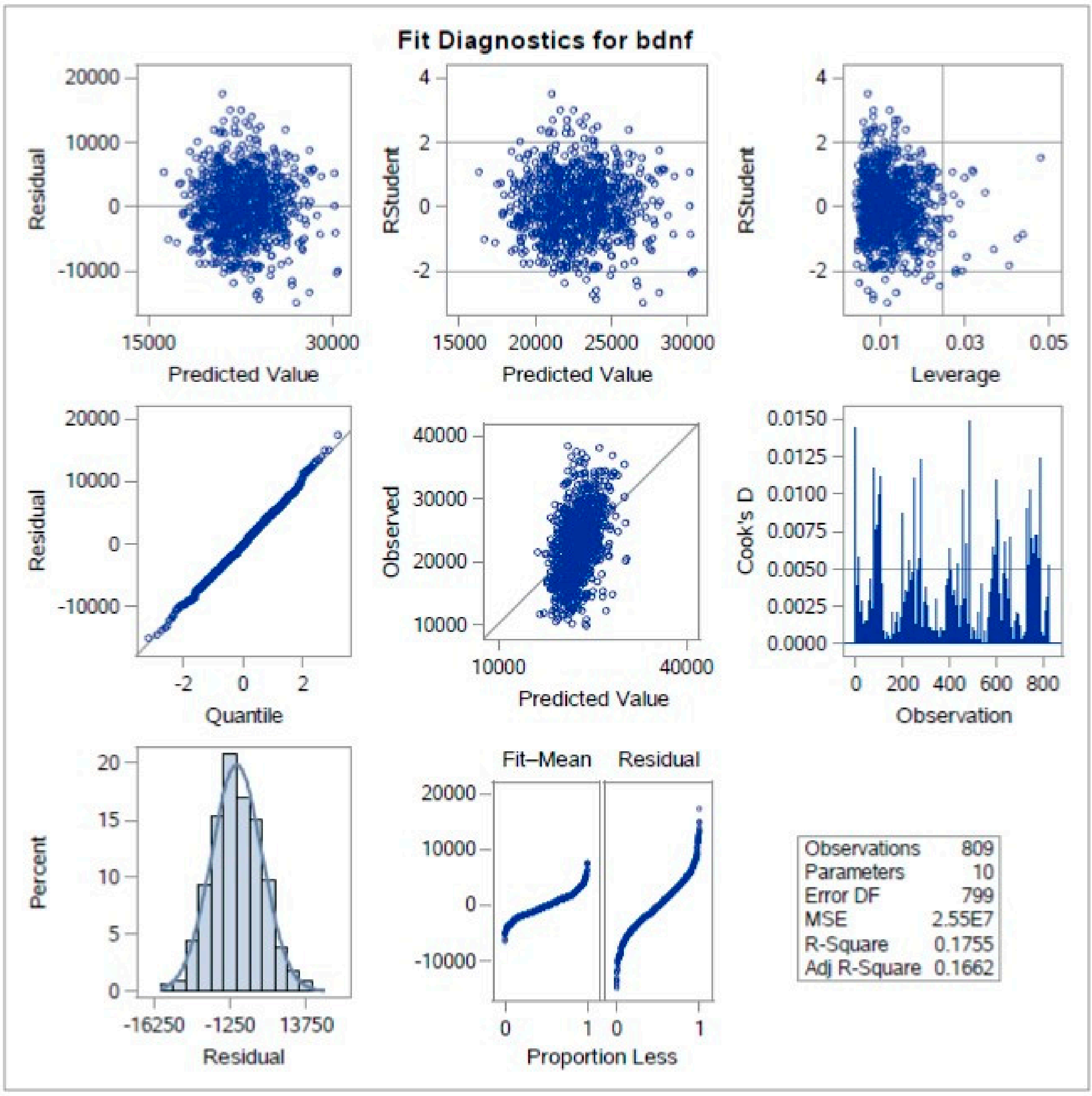

Suppl. Figure 9 – Fit diagnostics for the association between BDNF and VO_2peak in females with additional adjustment for platelets. Residual vs. predicted value, RStudent vs. predicted value, Rstudent vs. leverage, residual vs. quantile, predicted vs. observed, Cook's D and residual distribution plots.

Suppl. Figure 10 – Fit diagnostics for the association between BDNF and VO_2peak/kg in males with additional adjustment for platelets.

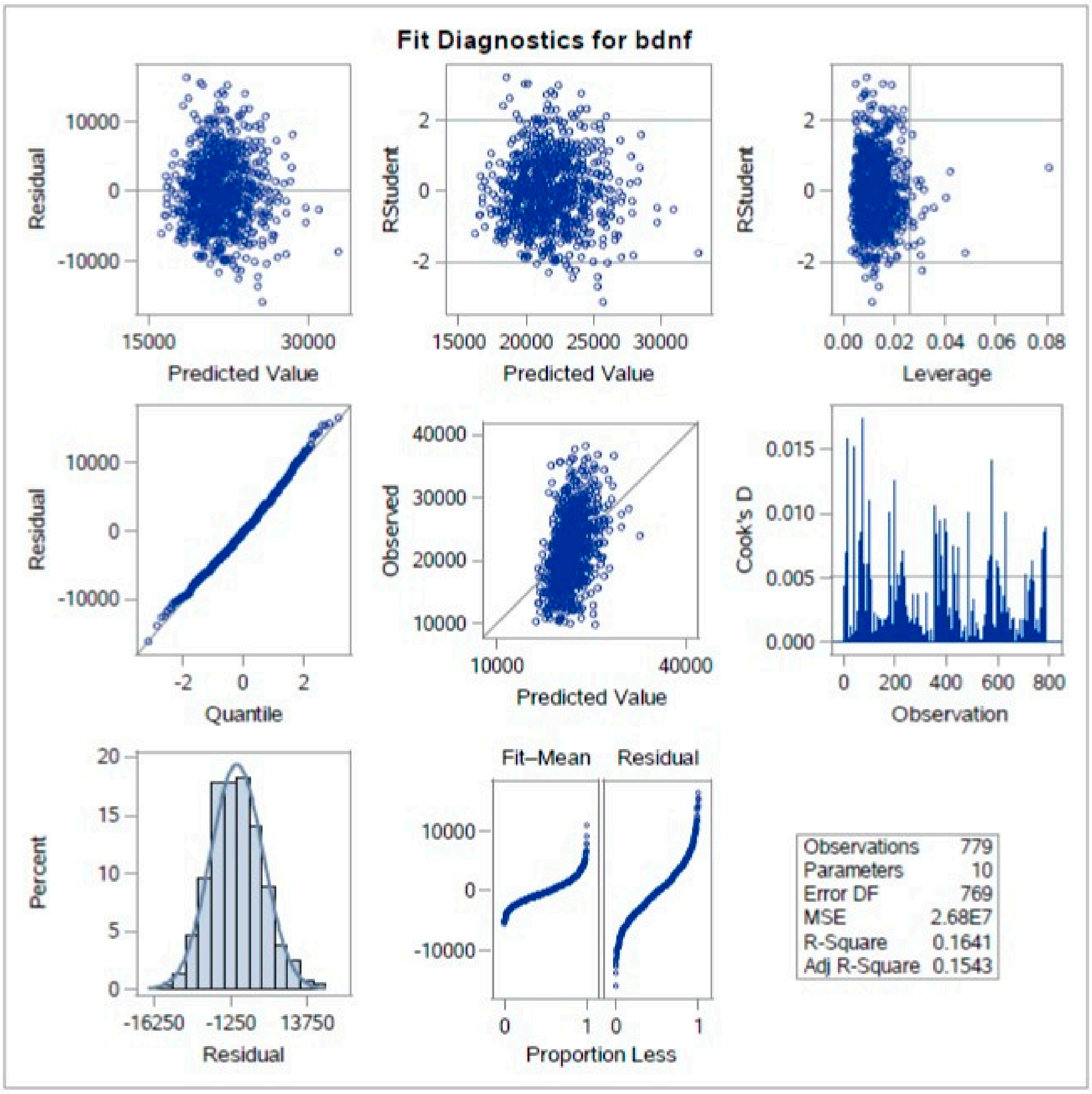

Suppl. Figure 10 – Fit diagnostics for the association between BDNF and VO_2peak/kg in males with additional adjustment for platelets. Residual vs. predicted value, RStudent vs. predicted value, Rstudent vs. leverage, residual vs. quantile, predicted vs. observed, Cook's D and residual distribution plots.

Suppl. Figure 11 – Fit diagnostics for the association between BDNF and VO_2peak/kg in females with additional adjustment for platelets.

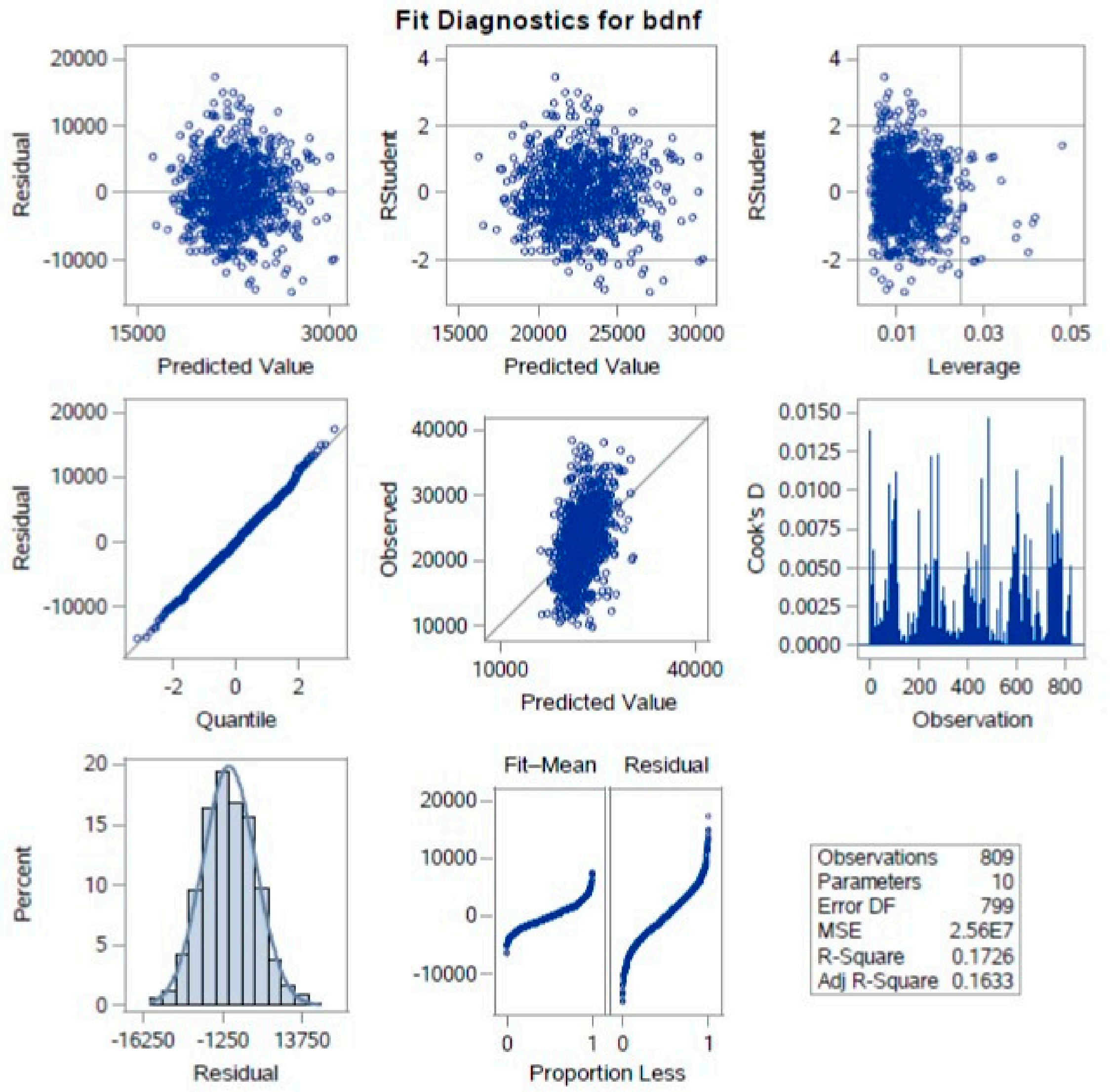

Suppl. Figure 11 – Fit diagnostics for the association between BDNF and VO_2peak/kg in females with additional adjustment for platelets. Residual vs. predicted value, RStudent vs. predicted value, Rstudent vs. leverage, residual vs. quantile, predicted vs. observed, Cook's D and residual distribution plots.

Suppl. Figure 12 – Fit diagnostics for the association between BDNF and VO_2@AT in males with additional adjustment for platelets.

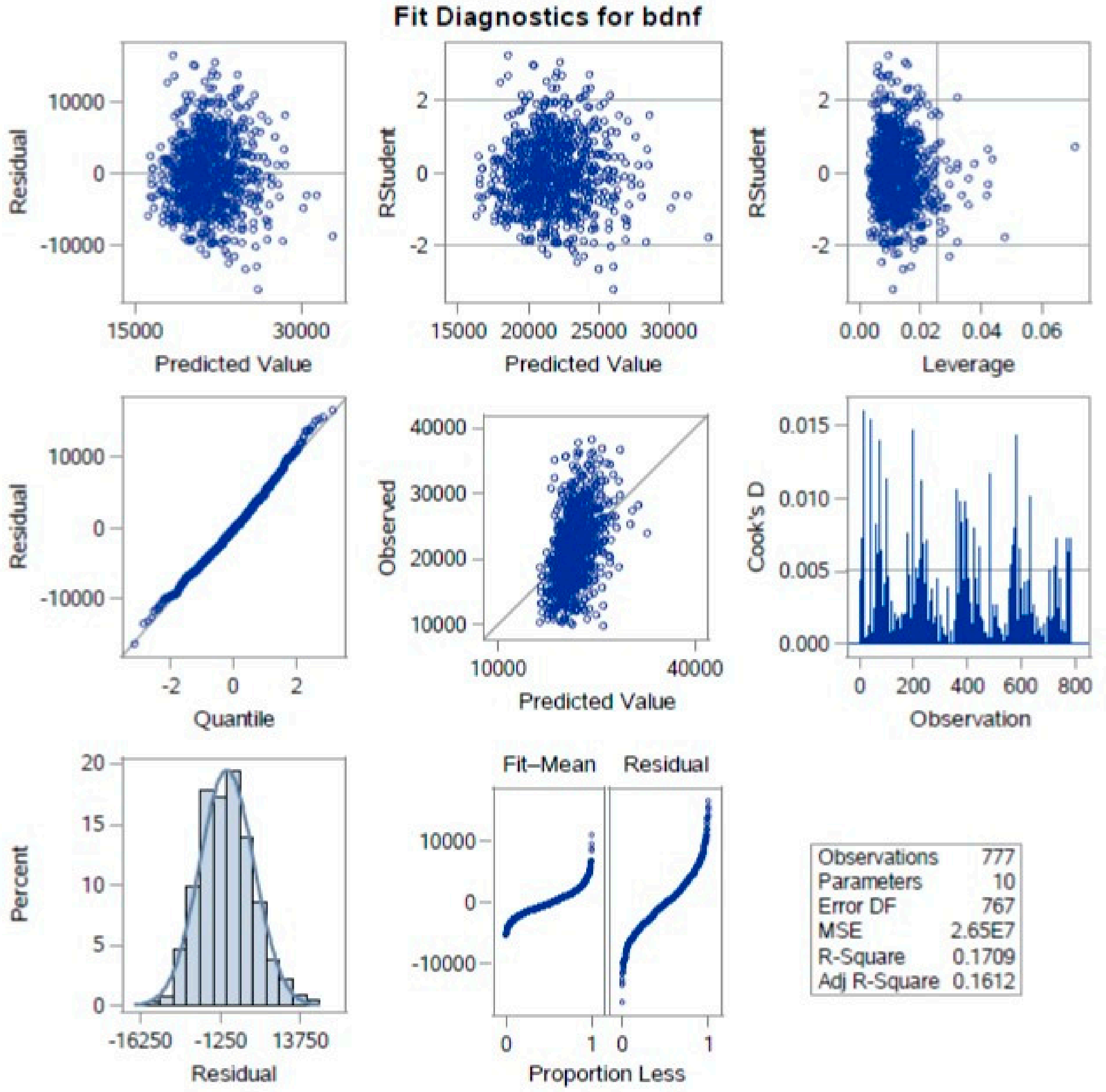

Suppl. Figure 12 – Fit diagnostics for the association between BDNF and VO_2@AT in males with additional adjustment for platelets. Residual vs. predicted value, RStudent vs. predicted value, Rstudent vs. leverage, residual vs. quantile, predicted vs. observed, Cook's D and residual distribution plots.

Suppl. Figure 13 – Fit diagnostics for the association between BDNF and VO_2@AT in females with additional adjustment for platelets.

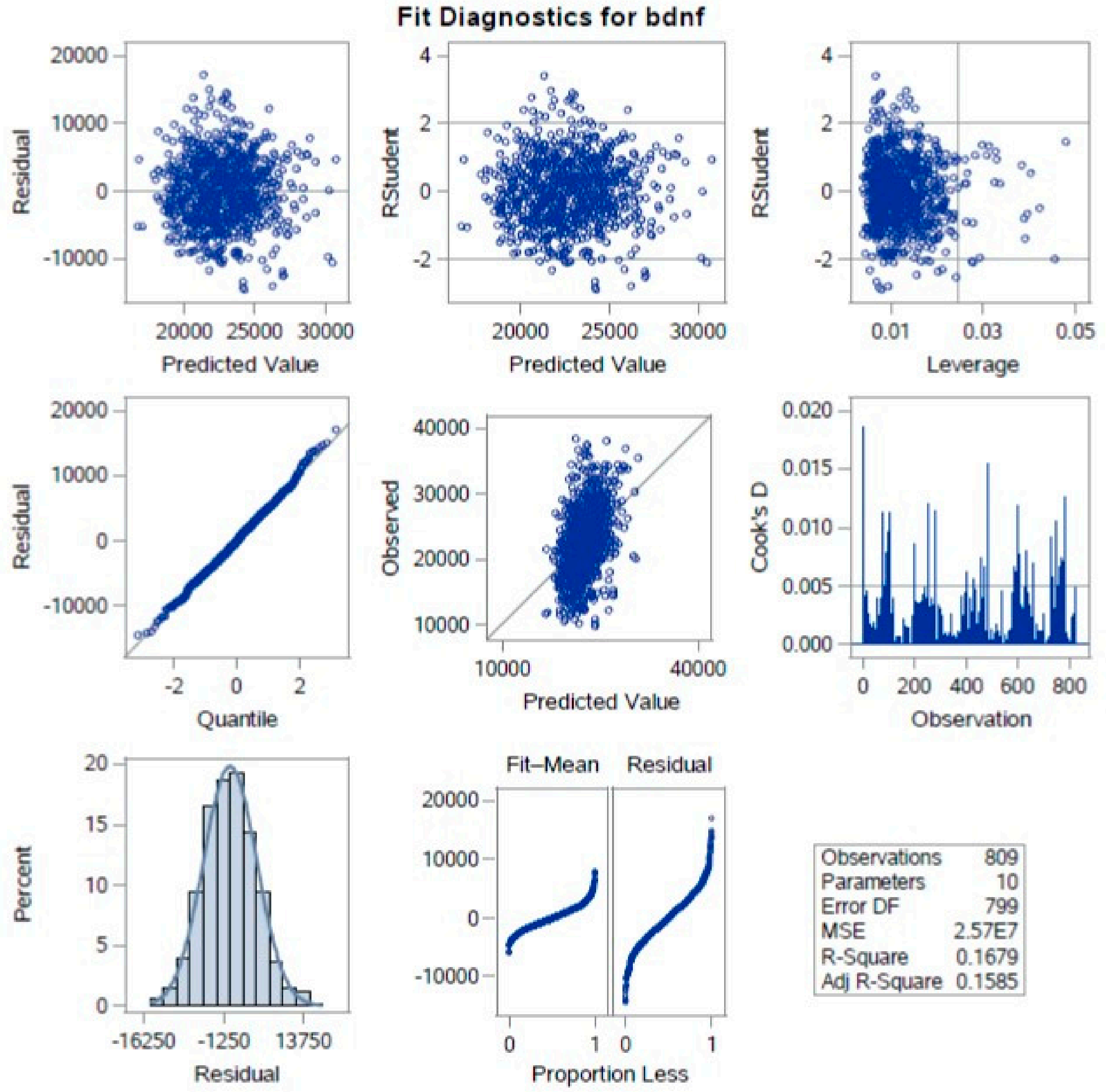

Suppl. Figure 13 – Fit diagnostics for the association between BDNF and VO_2@AT in females with additional adjustment for platelets. Residual vs. predicted value, RStudent vs. predicted value, Rstudent vs. leverage, residual vs. quantile, predicted vs. observed, Cook's D and residual distribution plots.
